# Neonatal* Streptococcus pneumoniae* Pneumonia Induces an Aberrant Airway Smooth Muscle Phenotype and AHR in Mice Model

**DOI:** 10.1155/2019/1948519

**Published:** 2019-01-06

**Authors:** Xin Peng, Yi Wu, Xiao Kong, Yunxiu Chen, Yonglu Tian, Qinyuan Li, Xiaoyin Tian, Guangli Zhang, Luo Ren, Zhengxiu Luo

**Affiliations:** ^1^Key Laboratory of Pediatrics in Chongqing, China; ^2^Department of Children's Hospital of Chongqing Medical University of Education, Key Laboratory of Child Development and Disorders, China; ^3^Department of Respiratory Medicine, Children's Hospital of Chongqing Medical University, Chongqing, China

## Abstract

Our previous study showed that neonatal* S. pneumoniae* infection aggravated airway inflammation and airway hyperresponsiveness (AHR) in an OVA-induced allergic asthma model. As airway smooth muscle (ASM) plays a pivotal role in AHR development, we aim to investigate the effects of neonatal* S. pneumoniae* pneumonia on ASM structure and AHR development. Non-lethal neonatal pneumonia was established by intranasally infecting 1-week-old BALB/C mice with the* S. pneumoniae* strain D39. Five weeks after infection, the lungs were collected to assess the levels of *α*-SMA and the contractile proteins of ASM. Our results indicate that neonatal* S. pneumoniae* pneumonia significantly increased adulthood lung *α*-SMA and SMMHC proteins production and aggravated airway inflammatory cells infiltration and cytokines release. In addition, the neonatal* S. pneumoniae* pneumonia group had significantly higher Penh values compared to the uninfected controls. These data suggest that neonatal* S. pneumoniae* pneumonia promoted an aberrant ASM phenotype and AHR development in mice model.

## 1. Introduction

Pneumonia is a common disease afflicting children, especially in developing countries [1].* Streptococcus pneumoniae (S. pneumoniae)* is the predominant bacteria responsible for community acquired pneumonia [2]. Epidemiological studies show that colonization of the neonatal respiratory tract with* S. pneumoniae*,* Haemophilus influenzae*, or* Moraxella catarrhalis* significantly increases the risk of asthma in the first 5 years of life [3, 4]. Asthma, characterized by chronic airway inflammation, airway hyperresponsiveness (AHR), and remodeling, is one of the most common chronic airway diseases among children worldwide [5, 6]. AHR, which is largely attributed by airway structural changes including airway smooth muscle (ASM) hypertrophy/hyperplasia, has long been considered a cardinal feature of asthma [7, 8]. Changes in structure and/or function of ASM have been observed in both asthma patients and experimental asthma models [9, 10]. Since ASM is responsible for airway constriction [11], alterations including hyperplasia/hypertrophy and/or dysregulation of contractile proteins of ASM can trigger AHR and airway remodeling [12, 13]. Our previous study stated neonatal* S. pneumoniae* infection aggravates airway inflammation and AHR in the ovalbumin (OVA) -induced allergic asthma model [14]. Whether neonatal* S. pneumoniae* infection induces asthma is associated with the alterations of ASM structure and/or function remains unclear. This intriguing observation promoted us to further investigate the effects of neonatal* S. pneumoniae* pneumonia on adulthood ASM structure and AHR development absent from allergen challenge. In this study, we found that neonatal* S. pneumoniae* pneumonia mice had significantly higher levels of alpha-smooth-muscle-actin (*α*-SMA) and smooth-muscle-myosin-heavy-chain (SMMHC) in adulthood lung tissues, as revealed by immunohistochemistry and Western blot analysis. The expression of smooth muscle-specific genes (actin-*α*2-smooth-muscle-aorta, Acta2 and myosin-heavy-chain-11-smooth-muscle, Myh11, respectively) was remarkably increased in neonatal* S. pneumoniae* pneumonia group, consistent with the Western blotting and histologic results. Furthermore, AHR was also remarkably higher in the* S. pneumoniae* pneumonia group compared to the controls. Taken together, neonatal* S. pneumoniae* pneumonia promoted an aberrant ASM phenotype and AHR development absent from allergen challenge in mice model.

## 2. Materials and Methods

### 2.1. Establishment of the Non-Lethal Neonatal Pneumonia Model Using S. pneumoniae

All experimental protocols were approved by the Institutional Animal Care and Research Advisory Committee of the Chongqing Medical University. The animals were treated in accordance with the guidelines issued by the Chinese Council on Animal Care. Parturient BALB/C mice were purchased from Animal Resources Centre of Chongqing medical university, housed separately, and closely monitored for births. Newborn mice were housed at 25°C under a 12 h light/dark cycle and provided with adequate food and water. We established a non-lethal neonatal* S. pneumoniae* model of pneumonia in these neonates as described in our previous study [14]. Briefly,* S. pneumoniae* D39 strain was inoculated into tryptic soy broth (Pangtong, China) and cultured for 12-14h at 37°C under 5% CO_2_. Neonatal (1-week-old) BALB/C mice were infected intra-nasally with 2 × 10^6^ colony-forming units (CFU) of* S. pneumonia* in 5 *μ*l phosphate buffered saline (PBS), while the mock-infected controls received the same volume of sterile PBS. Five weeks postinfection, the lungs were removed and homogenized, and the tissue homogenates were cultured on blood agar for 24 h (at 37°C under 5% CO_2_) to determine the bacterial load.

### 2.2. Histology of the Lungs

Five weeks after pneumonia, i.e., when the neonates had reached adulthood, the mice were euthanized with a lethal dose of 10% chloralhydrate (0.3ml/100g, intraperitoneally), and their lungs were harvested. The left lungs were desiccated and embedded in paraffin after fixing in 4% paraformaldehyde. Tissue sections (4*μ*m thick) were stained with hematoxylin and eosin (H&E; Sigma-Aldrich), and the lung lesions were semiquantitatively scored as described previously [15]: 0 points for no cell; 1 point for few cells; 2 points for a ring of inflammatory cells 1 cell layer deep; 3 points for a ring of inflammatory cells 2 to 4 cells deep; 4 points for a ring of inflammatory cells of >4 cells deep.

### 2.3. Bronchoalveolar Lavage Fluid (BALF) Analysis

Following euthanization of the mice as described above, the BALF was obtained by washing the lungs five times with 1 ml ice-cold PBS. The number of total cells, differential cell counts, and the level of cytokines including IL-4, IL-5, IL-13, IL-17A, and INF-*γ* were determined as previously described [14]. The cytokine levels were determined using specific enzyme-linked immunosorbent assay (ELISA) kits (Neobioscience, Shenzhen, China) according to the manufacturer's instructions.

### 2.4. Immunohistochemistry (IHC)

The lung tissue sections were deparaffinized and dehydrated as per standard protocols. After blocking endogenous peroxidase activity and non-specific staining with H_2_O_2_ and 5% bovine serum albumin (BSA), respectively, the sections were incubated with mouse monoclonal anti-*α*-SMA, rabbit monoclonal anti-SMMHC, or rabbit monoclonal anti-smooth muscle 22 alpha (SM22*α*) antibodies (all diluted 1:100, Sigma-Aldrich, St. Louis, MO) at 4°C for 12 h. The sections were washed with PBS and incubated with secondary antibody for 30 min at 37°C. The positive signals were developed using the 3,3′-diaminobenzidine (DAB) chromogen, and the sections were counterstained with hematoxylin. The basement membrane perimeter (Pbm) and the *α*-SMA^+^, SMMHC^+^, and SM22*α*^+^ areas [16] were outlined and analyzed with the Image-Pro Plus 6.0 software (Image-Pro® 6.0, USA). For accurate morphometric quantification, Pbm^2^ was first used to standardize the region of interest, i.e., sections of the airway wall and connective tissue attachments excluding the blood vessels [16–18]. The *α*-SMA^+^ area/Pbm^2^, SMMHC^+^ area/Pbm^2^ and SM22*α*^+^ area/Pbm^2^ ratios were calculated to, respectively, define the *α*-SMA^+^ area, SMMHC^+^ area, and SM22*α*^+^ area around the airway [17, 19].

### 2.5. Western Blotting

Total protein was extracted from the harvested lungs of adult mice and denatured with the SDS-PAGE loading buffer. Equal amounts of protein from each group were separated on 10% SDS-PAGE and then transferred onto polyvinylidene difluoride (PVDF) membranes (Millipore). The membranes were incubated with mouse anti-*α*-SMA (1:500; Sigma), rabbit anti-SMMHC (1:1000; Sigma), rabbit anti-SM22*α* (1:1000; Sigma), or rabbit anti-GAPDH (1:1000; Proteintech) antibody for 12 h at 4°C. After washing with PBS, the membranes were incubated with the secondary antibody, and the positive bands were quantified using Quantity-One software relative to GAPDH.

### 2.6. Real-Time Quantitative PCR (RT-qPCR)

Total RNA was extracted from the lungs of adult mice with TRIzol (Invitrogen, CA) and reverse transcribed into cDNA using the PrimeScript RT kit (TaKaRa, Japan). The relative expressions of *α*-SMA gene (Acta2), SMMHC gene (Myh11), and SM22*α* gene (transgelin; Tagln) were detected using the RT-qPCR assay kit from Life Technologies. The sequences of the respective forward (F) and reverse (R) primers are as follows:

  Acta2-F 5′-TGCTGGACTCTGGAGATGGTGTG-3′
  Acta2-R 5′-CGGCAGTAGTCACGAAGGAATAGC-3′
  Myh11-F 5′-CCATTGCCGACACAGCCTACAG-3′
  Myh11-R 5′-GGATGCCACCACAGCCAAGTAC-3′
  Tagln-F 5′-AGATGGAACAGGTGGCTCAATTCTTG-3′
  Tagln-R 5′- CCTTCATAGAGGTCAACAGTCTGGAAC-3′
  GAPDH-F 5′-CAGCGACACCCACTCCTCCACCTT-3′
  GAPDH-R 5′- CATGAGGTCCACCACCCTGTTGCT -3′.

### 2.7. Airway Hyperresponsiveness (AHR)

Five weeks after pneumonia, airway hyperresponsiveness (AHR) was determined by whole-body plethysmograph (Emka instrument; France) as previously described [20, 21]. Briefly, conscious and spontaneously breathing mice were exposed to aerosolized normal saline followed by increasing concentrations of aerosolized methacholine (3.125, 6.25, 12.5, 25, and 50 mg/ml; Sigma, USA) in normal saline for 3 min. The highest enhanced pauses (Penh) value obtained during each methacholine challenge was expressed as a proportion of the basal Penh value in response to normal saline challenge. Penh is a dimensionless value which reflects airway hyperresponsiveness. It represents a function of the ratio of peak expiratory flow to peak inspiratory flow and a function of the timing of expiration.

### 2.8. Statistical Analysis

Data were expressed as means ± SD. One-way analysis of variance (ANOVA) and Student's* t*-tests were used to compare the groups. All statistical analyses were performed using Graph Pad Prism (version 5.0; Graph Pad, La Jolla, CA, USA) and p<0.05 was considered statistically significant.

## 3. Results

### 3.1. Duration and Severity of Neonatal Pneumonia Caused by* S. pneumoniae *Infection

**Figure 1:**
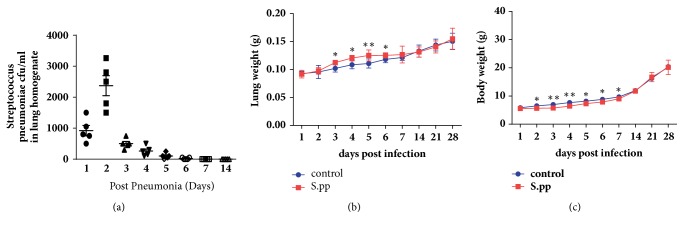
**Neonatal* S. pneumoniae* pneumonia affects lung bacterial load and lung and body weight.** (a)* S. pneumoniae *colony counts in the lung. (b) Lung weight. (c) Body weight. *∗*P<0.05, *∗∗*P<0.01, compared to the control group.* S.pp: S. pneumoniae* pneumonia.

To determine the duration and severity of neonatal pneumonia, the pulmonary bacterial load and the lung and body weights of the mice were assessed.* S. pneumoniae *were cleared from the lungs within 7 days of infection. While the lung and the total body weight increased in the uninfected control mice with age, the* S. pneumoniae* infected mice lost a significant amount of body weight within the first week post-infection, although their lungs were heavier compared to the control group 3 to 6 days post-infection (Figures 1(a)–1(c)). After 7 days of initiating pneumonia, the lung and body weights of the infected mice were restored to that of the uninfected mice. These results indicated that neonatal* S. pneumoniae* pneumonia had minimal effects on lung bacterial load and lung and body weight of adult mice.

### 3.2. Neonatal* S. pneumoniae *Pneumonia Altered Airway Smooth Muscle Productions in Mice Model

**Figure 2:**
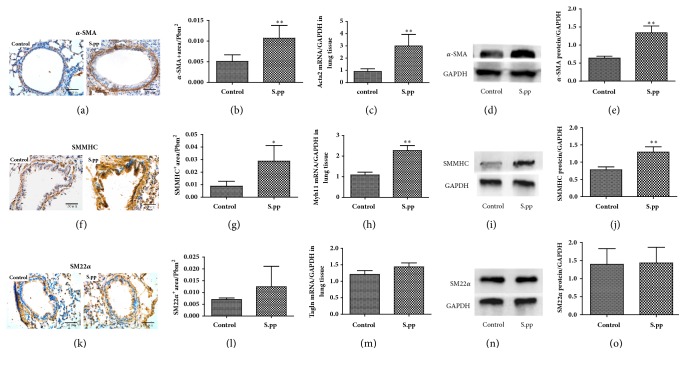
**Neonatal* S. pneumoniae* pneumonia alters airway smooth muscle productions in mice model.** IHC staining of lung tissues from both mock-infected (control) and neonatal* S. pneumoniae* pneumonia mice (S.pp) showing the* in situ* expression of *α*-smooth muscle actin (*α*-SMA) (a), smooth muscle myosin heavy chain (SMMHC) (f), and smooth muscle 22 alpha (SM22*α*) (k) (400x magnification). The relative *α*-SMA-positive (*α*-SMA^+^ area/Pbm^2^) (b), SMMHC-positive (SMMHC^+^ area/Pbm^2^) (g), and SM22*α*-positive (SM22*α*^+^ area/Pbm^2^) (l) areas are also shown. RT-qPCR was used to analyze Acta2-mRNA (c), Myh11-mRNA (h), and Tagln-mRNA (m) levels in the lung tissues. The *α*-SMA (d, e), SMMHC (i, j), and SM22*α* (n, o) protein levels in the lung tissues were analyzed by Western blotting. All data are presented as means ± SD. (n =5/group). *∗∗*P<0.01, compared to the control group.

To determine the effects of neonatal* S. pneumoniae* pneumonia on airway smooth muscle productions, we analyzed the expression of *α*-SMA and the contractile proteins of ASM (SMMHC and SM22*α*) in adulthood lung tissues. Neonatal* S. pneumoniae* pneumonia significantly increased the relative *α*-SMA positive area (*α*-SMA^+^ area/Pbm^2^) (0.01071 ± 0.003081* vs* 0.005089 ± 0.001586, P<0.01, Figures 2(a) and 2(b)), Acta2-mRNA (2.995 ± 0.9433* vs* 0.9065 ± 0.2148, P<0.01, Figure 2(c)), and *α*-SMA protein levels (1.338 ± 0.5061* vs* 0.6376 ± 0.1443, P<0.01, Figures 2(d) and 2(e)) in the adult lungs compared to the uninfected controls. In addition, the relative SMMHC positive area (0.02875 ± 0.01251* vs* 0.008826 ± 0.003849, P<0.05), Myh11-mRNA (2.270 ± 0.5375* vs* 1.084 ± 0.3131, P<0.01), and SMMHC protein levels (1.291 ± 0.4872* vs* 0.7768 ± 0.2793, P<0.01) were also significantly higher in the* S. pneumoniae* pneumonia group compared to the uninfected controls (Figures 2(f)–2(j)). In contrast, the relative SM22*α* positive area, Tagln-mRNA, and SM22*α* protein levels were similar in both groups (Figures 2(k)–2(o)). These results indicated neonatal* S. pneumoniae* pneumonia induced an increase in airway smooth muscle mass and airway remodeling in mice model.

### 3.3. Neonatal* S. pneumoniae* Pneumonia Aggravated Airway Inflammatory Cells Infiltration and Cytokines Release in Mice Model

**Figure 3:**
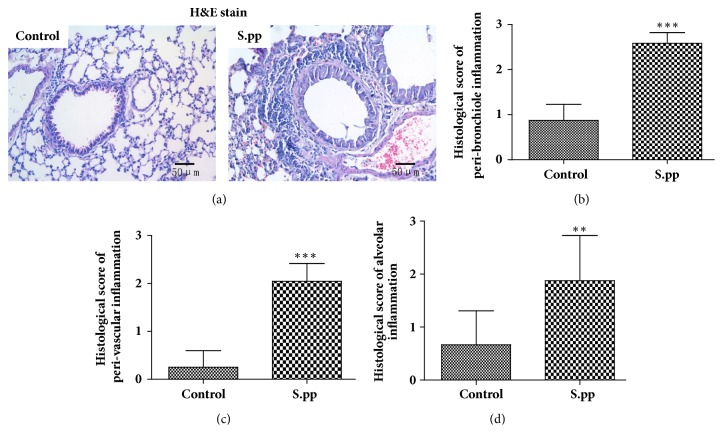
**Neonatal* S. pneumoniae* pneumonia increases adulthood inflammatory cell infiltration in the lung tissues.** H&E staining of lung samples from mock-infected and neonatal* S. pneumoniae *pneumonia mice 5 weeks after pneumonia (a) (200x magnification). Histological scores of pulmonary peri-bronchiolitis (b), pulmonary perivasculitis (c), and pulmonary alveolitis (d). Data are shown as mean ± SD (n=6-8 mice/group). *∗∗*P<0.01, *∗∗∗*P<0.001, compared to the control group.

Five weeks after pneumonia, the lung tissues and BALF were collected to assess airway inflammation. The neonatal* S. pneumoniae* pneumonia mice had significantly higher infiltration of inflammatory cells compared to the controls (Figure 3(a)). The inflammation scores for pulmonary peri-bronchiolitis (2.584 ± 0.2379* vs* 0.8738 ± 0.3544, P<0.001), pulmonary peri-vasculitis (2.043 ± 0.3740* vs* 0.2513 ± 0.3468, P<0.001), and pulmonary alveolitis (1.876 ± 0.8537* vs* 0.6650 ± 0.6412, P<0.01) in the infected mice were remarkably increased compared to the controls (Figures 3(b)–3(d)).

Consistent with the lung inflammation results, neonatal* S. pneumoniae* pneumonia mice had significantly higher counts of total inflammatory cells, neutrophils, macrophages, lymphocytes, and eosinophils in the BALF compared to controls (Table 1). In addition, the levels of IL-4, IL-5, IL-13, and IL-17A in BALF were also significantly higher in the pneumonia group (Table 2). Our results indicate neonatal* S. pneumoniae* pneumonia aggravated adulthood airway inflammatory cells infiltration and cytokines release.

### 3.4. Neonatal* S. pneumoniae* Pneumonia Promoted AHR Development in Mice Model

**Figure 4:**
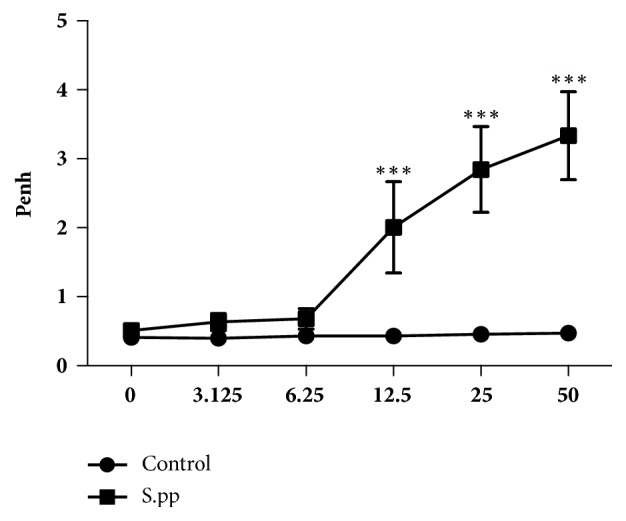
**Neonatal* S. pneumoniae* pneumonia promotes AHR development in adult mice.** Five weeks after pneumonia, whole-body plethysmography was conducted in mock-infected (control) and neonatal* S. pneumoniae* pneumonia mice (S.pp) with methacholine. All data are presented as mean ± SD (n=6-8 mice/group). *∗∗∗* P<0.001, compared to the control group.

Five weeks after neonatal* S. pneumoniae* pneumonia, AHR was assessed by the calculation of Penh values (i.e., enhanced respiratory pausing). No significant differences were found in the baseline of airway responsiveness of both groups following normal saline challenge. With the increase of the concentration of methacholine, the airway responsiveness of the* S. pneumoniae* pneumonia group was significantly higher compared to the controls when exposed to 12.5 mg/ml (2.005 ± 0.6622* vs* 0.4327 ± 0.03387, P<0.001), 25 mg/ml (2.845 ± 0.6230* vs* 0.4580 ± 0.02559, P<0.001), and 50.0 mg/ml (3.335 ± 0.6364* vs* 0.4714 ± 0.04637, P<0.001) methacholine (Figure 4). Therefore, neonatal* S. pneumoniae* pneumonia promoted AHR development in mice model.

## 4. Discussion

Neonatal* S. pneumoniae* infection promotes experimental asthma development in adults [14]. While Preston et al. [22] stated adulthood* S. pneumoniae* infection protected against allergic asthma in mice model. Al-Garawi et al. [23] showed that neonatal exposure to allergens (HMD) in the presence of acute influenza virus infection induced lung remodeling and imprinted an asthmatic phenotype in adult BALB/C mice. Horvat et al. [24, 25] found that neonatal, and not adult, respiratory* Chlamydia pneumoniae* infections altered lung function and structure and enhanced the severity of respiratory allergies in later life in mice. Whether neonatal* S. pneumoniae* infection promotes allergic asthma by inducing airway remodeling and AHR in the absence of allergens is not completely clarified. Here we showed for the first time in mice model that neonatal* S. pneumoniae* pneumonia increased the expression of *α*-SMA in the airway, partially altered the ASM phenotype, and induced airway inflammation and AHR.

ASM is the main structural component of the airway structure and controls the diameter of bronchi and bronchioles via contractile function [11]. ASM remodeling, in the form of ASM cells hypertrophy, hyperplasia, and changes in contractility, is associated with AHR and airway remodeling [26]. Viruses such as* rhinovirus*,* influenza*, and* respiratory syncytial virus* can promote ASM hyperplasia and enhance the contractility and secretory functions of ASM [27–29].* C. pneumoniae* has been shown to induce ASM proliferation* in vitro* via the increased secretion of IL-6, PDGF, and FGF [30]. However, few studies have investigated the effects of bacterial infection on ASM.

To the best of our knowledge, this is the first study to explore the effects of neonatal* S. pneumoniae* infection on the expression of airway *α*-SMA and ASM contractile proteins in adults. We found that neonatal* S. pneumoniae* pneumonia upregulated airway Acta2 and Myh11 mRNAs and *α*-SMA and SMMHC proteins in the adulthood mice and also promoted AHR. Our results are consistent with previous studies which correlated airway structural changes to AHR development [31–33]. The remodeling and contractile capacity of ASM can be enhanced by inflammatory mediators further to contribute to AHR [34]. Our data showed that neonatal* S. pneumoniae* pneumonia can increase the infiltration of inflammatory cells, both in the lung tissues and in the BALF, along with releasing higher levels of the pro-inflammatory cytokines (IL-4, IL-5, IL-13, and IL-17A). Type II (Th2) cytokines, including IL-4, IL-5, and IL-13, can promote AHR, airway remodeling, and mucus hypersecretion [35]. IL-4 enhances ASM contractility and proliferation of the ASM cells [36]. IL-13 also promotes ASM contraction and remodeling and thus leads to AHR via calcium accumulation [37]. Finally, IL-17A not only promotes ASM cell proliferation and contraction, but also induces the secretion of pro-AHR cytokines and chemokines [29, 38]. We found that neonatal* S. pneumoniae* pneumonia significantly increased the levels of AHR, IL-4, IL-5, IL-13, and IL-17A relative to controls, which is consistent with the above studies. Therefore, in this study, we speculate that the secretion of inflammation cytokines and chemokines may promote ASM remodeling and AHR of adult mice. The specific mechanism of action remains to be further studied.

As one of the contractile ASM proteins, SMMHC expression increased is highly involved in the enhancement of airway remodeling which contributes to AHR during chronic asthma in mice model [39]. Chronic inflammation can regulate the expression of smooth muscle-specific contractile proteins including SMMHC [40], while CD4^+^ T cells are known to induce SMMHC and promote AHR [41]. Consistent with this, neonatal* S. pneumoniae* infection promotes CD4^+^ T cells production in adulthood [14]. We found that neonatal* S. pneumoniae* infection significantly increased SMMHC levels, which may be an important factor for airway remodeling and AHR. Growing evidence indicates that airway inflammation and ASM remodeling also synergize with each other [17, 42]. Airway inflammation induced by dysfunctional ASM can promote SMMHC protein expressions, which can further aggravate AHR by enhancing ASM remodeling [43].

Our study also had some limitations. The invasive technique has been used widely to measure airway resistance and hyperresponsiveness. Other studies and our laboratory studies demonstrate that Penh can be used as an indicator of AHR [15, 44–50]. Here we only used Penh to represent airway responsiveness due to the high mortality with an invasive technique. On the other hand, there were a series of connections between ASM and inflammation. Some studies indicated that ASM remodeling triggers airway inflammation and AHR by producing cytokines [51, 52]. Another study showed that the cytokines, chemokines, and matrix proteins produced by the ASM cells were necessary for their* in vitro* proliferation [53]. In addition, airway inflammation triggered by dysfunctional ASM can further promote its contraction [54]. In this study, we did not investigate the direct link between ASM and inflammation; further studies are needed to clarify the direct link between ASM remodeling and airway inflammation induced by neonatal* S. pneumoniae* pneumonia.

## 5. Conclusions

In conclusion, neonatal* S. pneumoniae* pneumonia promotes airway smooth muscle phenotype and AHR in adult mice, which provides a theoretical basis for asthma prevention.

## Figures and Tables

**Table 1:** Neonatal *S. pneumoniae* pneumonia increases inflammatory cell accumulation in the BALF.

Group	n	Total cells	Neutrophils	Macrophage	Lymphocyte	Eosinophils
		(×10^5^)	(×10^4^)	(×10^4^)	(×10^4^)	(×10^4^)
Control	5	4.21 ± 0.765	6.85 ± 1.04	32.2 ± 4.74	8.49 ± 2.59	0.283 ± 0.0448
S.pp	5	16.8 ± 5.36*∗∗*	19.9 ± 5.17*∗∗*	57.4 ± 4.18*∗∗∗*	33.5 ± 11.8*∗∗∗*	1.1 ± 0.421*∗∗*

Data are shown as mean ± SD.

*∗∗*P<0.01, *∗∗∗* P<0.001, compared to the control group.

**Table 2:** Neonatal *S. pneumoniae* pneumonia increases cytokines production in BALF (pg·ml^−1^).

Group	n	IL-4	IL-5	IL-13	IL-17A	INF-*γ*
Control	6	20.5 ± 4.09	15.5 ± 7.04	12.4 ± 7.32	62.8 ± 11.38	44.9 ± 8.09
S.pp	6	29.1 ± 4.53*∗*	28.5 ± 11.6*∗∗*	21.7 ± 9.11*∗*	115.7 ± 10.45*∗∗∗*	19.8 ± 6.37*∗∗*

Data are shown as mean ± SD.

*∗*P<0.05, *∗∗*P<0.01, *∗∗∗* P<0.001, compared to the control group.

## Data Availability

The datasets used and/or analyzed during the current study are available from the corresponding author upon reasonable request.

## Abbreviations

Acta2: Actin-*α*2-smooth-muscle-aorta
AHR: Airway hyperresponsiveness
ASM: Airway smooth muscle
BALF: Bronchoalveolar lavage fluid
Myh11: Myosin-heavy-chain-11-smooth-muscle
PBS: Phosphate buffered saline
*S. pneumoniae*: * Streptococcus pneumoniae*
SM22*α*: Smooth-muscle-22-alpha
SMMHC: Smooth-muscle-myosin-heavy-chain
Tagln: Transgelin
*α*-SMA: Alpha smooth muscle actin.
